# Antimicrobial resistance at a turning point: microbial drivers, one health, and global futures

**DOI:** 10.3389/fmicb.2025.1698809

**Published:** 2025-11-17

**Authors:** Ayman Elbehiry, Eman Marzouk, Adil Abalkhail

**Affiliations:** Department of Public Health, College of Applied Medical Sciences, Qassim University, Buraydah, Saudi Arabia

**Keywords:** antimicrobial resistance (AMR), clinical microbiology, public health, diagnostic innovation, antimicrobial stewardship, global health policy, resistance surveillance

## Abstract

Antimicrobial resistance (AMR) is a major health threat of the 21st century, undermining the effectiveness of modern medical interventions and reversing decades of progress in infection control. Its drivers include microbial evolution, horizontal gene transfer, inappropriate use in human and veterinary medicine, agricultural practices, environmental reservoirs, and uneven regulation. This review integrates microbial, clinical, and environmental perspectives within a One Health framework. At the microbial level, resistance arises through mutation, gene transfer, and biofilm-associated tolerance, with soil, wastewater, and wildlife serving as conduits for spreading resistance elements. Advances in diagnostics—including matrix-assisted laser desorption/ionization time-of-flight mass spectrometry (MALDI-TOF MS), whole-genome sequencing (WGS), digital PCR, and CRISPR-based assays are transforming detection and surveillance, but deployment remains uneven, particularly in low- and middle-income countries. Antimicrobial stewardship now extends beyond hospitals, supported by decision support systems, artificial intelligence (AI), and community programs; however, gaps in surveillance capacity and policy implementation continue to limit impact. One Health linkages connect agricultural use, wastewater, and wildlife exposure with human risk, embedding clinical decisions within ecological and veterinary contexts. Persistent gaps include fragmented regulation, limited involvement of microbiologists in policy development, and weak incentives for antibiotic innovation. Priority directions include biomarker-guided prescribing, CRISPR-directed antimicrobials, microbiome-sparing therapeutics, and genomics-informed surveillance that integrates clinical and environmental data. Positioning the clinical microbiology laboratory as an operational hub can align rapid diagnostics, interpretive reporting, antimicrobial stewardship, and integrated surveillance (GLASS, EARS-Net, NARMS, and wastewater/wildlife monitoring) on a common platform. Clear reporting triggers and concise case vignettes can translate laboratory results into actionable bedside decisions and policy measures across diverse resource settings, with measurable benefits for patient outcomes and public health.

## 1 Introduction

The discovery of penicillin by Alexander Fleming in 1928 ushered in the antibiotic era and transformed infectious disease care. Procedures once fraught with risk, such as major surgery, organ transplantation, chemotherapy, and intensive care, became safer and far more feasible (Aminov, 2010). However, success had a downside. In human, veterinary, and agricultural settings, antibiotics were used too often, for too long, or without clear indication, and resistance followed (Ventola, 2015; Cassini et al., 2019). In 2019, antimicrobial resistance (AMR) was estimated to cause 1.27 million deaths directly and contribute to nearly 5 million more, a devastating global impact. Low and middle income countries (LMICs) shoulder the greatest burden, driven by limited diagnostic capacity and largely unregulated access to antimicrobials (Okeke et al., 2005; Mendelson and Matsoso, 2015; Murray et al., 2022).

At its core, AMR is microbial adaptation—mutation, horizontal gene transfer, and biofilm-linked tolerance shaped by pressures from hospital prescribing to antibiotic use in agriculture and contamination from pharmaceutical waste (Berendonk et al., 2015; Hernando-Amado et al., 2020). Because these pressures cut across people, animals, and environments, piecemeal fixes fail. What works is One Health: a joined-up view of shared ecosystems and shared risks (Robinson et al., 2016; Hoelzer et al., 2017). Within this system, clinical microbiologists generate the signals—pathogen identification, resistance profiles, trend alerts—that stewardship and surveillance depend on; when their input is sidelined, guidelines and policy suffer (Ashley et al., 2016; Friedman et al., 2016).

This review positions the clinical microbiology laboratory as the operational nexus that links rapid diagnostics and interpretive reporting to antimicrobial stewardship actions, integrated surveillance—World Health Organization (WHO) Global Antimicrobial Resistance and Use Surveillance System (GLASS), European Antimicrobial Resistance Surveillance Network (EARS-Net), and the National Antimicrobial Resistance Monitoring System (NARMS)—and One Health governance. It synthesizes evidence showing that pairing rapid organism identification and susceptibility testing with stewardship shortens time-to-effective therapy and reduces unnecessary broad-spectrum exposure (Perez et al., 2014; Reuter et al., 2019; Goshorn et al., 2023). It also maps laboratory “levers” (targeted/cascade reporting and diagnostic stewardship) to programmatic guidance from the Infectious Diseases Society of America (IDSA)/Society for Healthcare Epidemiology of America (SHEA) and the Centers for Disease Control and Prevention (CDC) Core Elements (Barlam et al., 2016; CDC, 2020).

Recent diagnostic advances—matrix-assisted laser desorption/ionization time-of-flight mass spectrometry (MALDI-TOF MS), real-time polymerase chain reaction (PCR), whole-genome sequencing (WGS), and metagenomics—have reshaped how laboratories detect pathogens and resistance determinants (Köser et al., 2014; Florio et al., 2020). Yet implementation remains uneven. WGS and metagenomics are invaluable for outbreak tracing and resistance surveillance but are constrained by cost, turnaround time, and the absence of standardized bioinformatics pipelines, as underscored by recent WHO GLASS and European Food Safety Authority (EFSA)/European Center for Disease Prevention and Control (ECDC) reports (Organization, W.H, 2022; ECDC, 2023). Meanwhile, outdated empiric therapy still dominates in many regions, perpetuating treatment failures and resistance (Omulo et al., 2015; Gandra et al., 2020). Agricultural antibiotic use also remains poorly regulated in large parts of the world, where non-therapeutic administration for growth promotion and prophylaxis selects for resistant bacteria along the food chain (Van Boeckel et al., 2015; Tang et al., 2017). Beyond clinical and agricultural settings, environmental reservoirs—including wastewater, surface water, soil, and the gut microbiome—sustain and disseminate resistance genes across microbial communities (Rizzo et al., 2013; Larsson et al., 2018; Dongre et al., 2025).

Despite extensive literature on the burden and drivers of antimicrobial resistance (AMR), many reviews emphasize biomedical or epidemiological narratives while overlooking the practical bridge between laboratory data, stewardship, and governance. This review addresses that gap. It integrates microbial mechanisms with One Health dissemination, highlights diagnostic innovation and stewardship strategy, and surfaces translational challenges. Uniquely, it frames microbiologists not only as technical experts but as essential actors who can connect science to policy and power equitable, evidence-informed responses to the AMR crisis.

In this shared framework, clinical microbiologists generate and interpret organism–mechanism signals; epidemiologists translate trends into actionable risk thresholds; veterinary services coordinate farm-to-fork controls; environmental agencies manage wastewater and catchment risks; and policymakers align reporting, formularies, procurement, and discharge standards with those signals.

## 2 Microbial foundations of AMR

**Figure 1:**
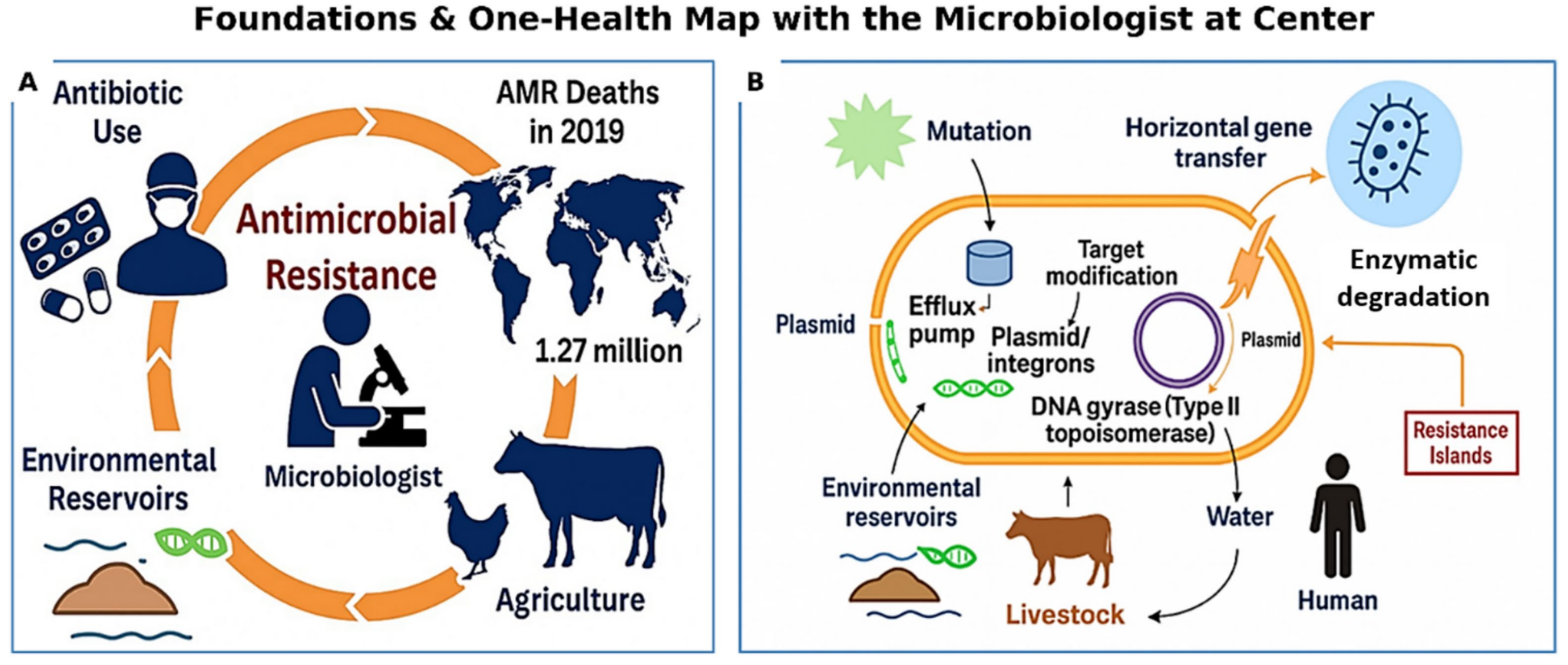
Foundations and One Health map with the clinical microbiologist at the center. **(A)** The One Health drivers of AMR—human antibiotic use, agriculture/livestock practices, and environmental reservoirs—set the stage for today’s crisis. For scale, the estimated global mortality burden in 2019 is shown (1.27 million deaths; Murray et al., 2022). **(B)** The core microbial playbook behind resistance: mutation and target modification; enzymatic drug inactivation (e.g., β-lactamases); multidrug efflux pumps; porin loss; mobile elements (plasmids, integrons); and genomic resistance islands. Arrows indicate cross-sector flow between environment, livestock, and humans. This panel is a conceptual overview; Table 1 lists representative exemplars and clinical implications, and cross-sector transmission pathways appear once in Figure 3.

AMR is microbial adaptation under antibiotic pressure—genetic, physiological, and ecological forces that play out across human, animal, and environmental domains. Grasping these foundations enables better diagnostics, surveillance, and stewardship. For concise mechanism definitions referenced throughout, see Figure 1B and Table 1.

**Table 1:** One Health drivers of AMR: key pathways, genetic mechanisms, and representative evidence.

Sector / driver	How it contributes to AMR	Example genes / mechanisms	Key references
Human clinical use & misuse	High and rising use plus non-prescription/OTC access (notably in LMICs) selects resistant pathogens.	ESBLs (e.g., *blaCTX-M*); carbapenemases (*blaNDM*, *blaKPC*).	Li et al. (2023); Klein et al. (2024)
Agriculture & livestock	Therapeutic/prophylactic/metaphylactic use selects resistant bacteria and plasmids that move along the food chain.	*mcr-1* (colistin); ESBLs (e.g., *blaCTX-M*); AmpC.	ECDC (2023); Garcias et al. (2024)
Treated-wastewater irrigation	Enriches ARGs in irrigated soils → transfer along soil→plant→invertebrates; runoff seeds surface waters.	*sul1*, *sul2*, *tetM*, *qnrS*, *blaCTX-M*, *blaTEM*, *intI1*.	Martínez et al. (2015); Karkman et al. (2018); Hendriksen et al. (2019)
Plant–soil interface (rhizosphere/phyllosphere/endosphere)	HGT in the rhizosphere; carriage on edible tissues → food-chain exposure.	*tetM*, *sul1*, *qnrS*, *blaCTX-M*, *ermB*, *intI1*.	Martínez et al. (2015)
Aquaculture	Antibiotics in fish/shrimp systems drive selection in water/sediments; ARGs spread through aquatic food webs.	*qnr*, *tet(A/B)*, *sul1/2/3*, *floR*.	Schar et al. (2020); Okeke et al. (2022); Milijasevic et al. (2024)
Wastewater (municipal & hospital)	WWTP influent/effluent carries antibiotics, ARGs/MGEs; plants can act as mixing/selection hubs if not optimized.	Diverse ARGs; class 1 integrons; carbapenemase genes.	Conforti et al. (2025); Punch et al. (2025)
Pharmaceutical manufacturing effluents	Hotspots with very high drug levels drive intense selection and downstream ARG enrichment.	Fluoroquinolone residues (e.g., ciprofloxacin) at mg/L levels.	Larsson et al. (2007); Mansouri et al. (2021)
Soil application of manure & biosolids	Land application introduces antibiotics, ARGs, and MGEs; increases ARG abundance/diversity; potential plant uptake.	*tet*, *sul*, *erm*; class 1 integrons.	Zhang et al. (2022); D’Angelo (2023); Wang et al. (2024b)
Wildlife vectors (e.g., gulls, urban birds)	Exposure at landfills/WWTPs/urban waters → carriage and long-distance dissemination of resistant bacteria.	ESBL *E. coli* (e.g., *blaCTX-M*), *qnr*, *tet*, *sul*; MDR *Salmonella*.	Zeballos-Gross et al. (2021); Fuentes-Castillo et al. (2023); Cagnoli et al. (2024)

### 2.1 Genetic basis of resistance: mutations and horizontal gene transfer

Bacteria acquire resistance via spontaneous mutations and horizontal gene transfer (HGT). Chromosomal changes alter drug targets, regulatory circuits, or membrane permeability (e.g., *gyrA*/*parC* for fluoroquinolones; *rpoB* for rifampicin). The fastest spread comes from mobilized antimicrobial resistance genes (ARGs) on plasmids, transposons, and class 1 integrons that move across taxa (Figure 1B; Table 1). Co-selection is common—extended-spectrum *β*-lactamases (ESBLs) paired with *qnr* or *mcr* loci—linking genetic context to clinical choices (Table 1). Reports of single plasmids co-carrying carbapenemase and colistin resistance genes compress last-line options into highly transferable elements, underscoring the need for genomic surveillance.

### 2.2 Evolutionary origins and environmental reservoirs of resistance genes

Many determinants trace to environmental bacteria where they evolved as survival tools. Metagenomics reveals homology between genes in today’s pathogens and ancient determinants from soil antibiotic producers (e.g., *Streptomyces*; D’Costa et al., 2011; Crofts et al., 2017). Anthropogenic antibiotic use accelerates mobilization and enrichment of these genes in human-associated populations, highlighting the ecological roots of the clinical resistome and motivating surveillance that spans humans, animals, and environments and policies that lower selective pressure.

### 2.3 Molecular mechanisms of resistance

Enzymatic degradation/modification is widespread: ESBLs and carbapenemases such as *Klebsiella pneumoniae* carbapenemase (KPC), New Delhi metallo-*β*-lactamase (NDM), and OXA-48 oxacillinase (OXA-48) hydrolyze β-lactams (van Duin and Doi, 2017; Bush and Bradford, 2020). Target modification includes *gyrA*/*parC* mutations (fluoroquinolones) and erm-mediated 23S rRNA methylation (macrolides; Davies and Davies, 2010; Cattoir and Leclercq, 2013). Multidrug efflux—AcrAB–TolC in *Escherichia coli* (*E. coli*), MexAB–OprM in *Pseudomonas aeruginosa* (*P. aeruginosa*) reduces intracellular drug levels (Li and Nikaido, 2004; Blair et al., 2015). Reduced permeability via porin loss limits entry, a hallmark of carbapenem-resistant *Enterobacterales* (Delcour, 2009; Wu et al., 2019).

In practice, mechanisms are often inferred from phenotypes; pairing targeted assays with phenotypes speeds organism–mechanism matching and optimizes therapy (Figure 1B; Table 1). Based on the clinical implications, OXA-48–like carbapenemases may present with low minimum inhibitory concentrations (MICs) and be missed without targeted tests (Boyd et al., 2022; CLSI-M100, 2025). Efflux and porin loss shift *β*-lactam MICs upward; plasmid-borne ESBLs raise empiric coverage thresholds and trigger infection-control actions per expert rules (Allander et al., 2024). Pairing phenotype with targeted assays and stewardship shortens time to appropriate therapy and enables earlier de-escalation (Timbrook et al., 2016; Peri et al., 2024).

### 2.4 Biofilm formation and ecological niches of resistance

Biofilms embed cells in a protective matrix, restrict diffusion, and foster persisters metabolically quiescent cells that survive exposure and reseed infection (Lewis, 2007; Hall and Mah, 2017). This biology underlies chronic and device-associated infections (e.g., catheter, prosthetic joint, ventilator-associated pneumonia; Donlan, 2001; Flemming et al., 2016) but is underrepresented in antimicrobial stewardship frameworks focused narrowly on genetics.

Beyond the clinic, the gut microbiota is a vast ARG reservoir with mobilization potential into pathogens (Penders et al., 2013; Van Schaik, 2015); ESBLs and vancomycin resistance genes occur even in healthy individuals (Hu et al., 2016; Pehrsson et al., 2016). Environmental co-selectors—pharmaceutical residues, disinfectants, heavy metals—enrich resistant strains (Bengtsson-Palme et al., 2018; Karkman et al., 2018). Wastewater treatment plants, as dense selective ecosystems, facilitate HGT and novel combinations (Rizzo et al., 2013; Manaia, 2017). Livestock and poultry amplify multidrug-resistant (MDR) *Salmonella* spp., *Campylobacter* spp., and *E. coli*, with genetic evidence of transmission to humans (Nhung et al., 2016; Madec et al., 2017). Actionable implication: embed biofilm-aware diagnostics, debridement/device strategies, and source control into stewardship pathways to complement gene-focused approaches.

### 2.5 Emergence of difficult-to-treat organisms Harboring combinatorial determinants

Co-selection and co-location of multiple determinants on mobilizable plasmids, genomic islands, or integrative elements produce phenotypes refractory even to combinations and now feature in guidance for Gram-negatives (Tamma et al., 2024). In *E. coli*, convergence of carbapenemases with colistin resistance (e.g., *blaNDM* on IncX3 with *mcr-1* on IncX4/IncI2) is documented in hospitals and the food chain; single plasmids carrying *blaNDM-5* + *mcr-1.1* have been isolated from patients and retail chicken (Liu et al., 2024; Liao et al., 2025). Mosaic plasmids encoding dual carbapenemases (e.g., *blaNDM-5* + *blaOXA-181*) can evade detection when carbapenem MICs are deceptively low (Zuo et al., 2024). In *Acinetobacter baumannii*, chromosomal resistance islands—*Acinetobacter baumannii* (*A. baumannii*) resistance island (AbaR)/ *A. baumannii* genomic resistance island (AbGRI)—consolidate *blaOXA-23* and aminoglycoside-modifying enzymes, stabilizing resistance in high-risk clones (Naderi et al., 2023; Wang et al., 2024a). In *P. aeruginosa*, genomic islands and class 1 integrons encode metallo-*β*-lactamases (MBLs; *blaVIM*, *blaIMP*) in epidemic lineages—sequence type (ST) 235 and ST111 (Flores-Vega et al., 2025; Hu and Chua, 2025).

Carbapenem-resistant hypervirulent *Klebsiella pneumoniae* arises when virulence plasmids (e.g., pLVPK-like) co-integrate with *blaKPC*/*blaNDM* plasmids, often via insertion sequence 26 (IS26)/integrative and conjugative element of *K. pneumoniae* (ICEKp), as seen in ST11-capsular locus (KL) 64 outbreaks (Tian et al., 2023; Chen et al., 2024; Chen et al., 2025). These architectures compress therapeutic options and complicate detection; low-level OXA-48–like activity is easily missed without targeted screening. Evolving toward genomic-context analysis—hybrid/long-read sequencing, assays for cryptic determinants, and plasmid/clone-level tracking—enables actionable organism–mechanism matching aligned with difficult-to-treat resistance (DTR) management (Tamma et al., 2024).

According to managemental implications, when genomic context suggests DTR risk (e.g., co-located carbapenemase + colistin resistance), laboratories should trigger reflex carbapenemase testing, infection-control alerts, and stewardship consults, with empiric regimens narrowed or escalated once mechanisms are confirmed (Table 1). For navigation, Table 1 captures major mechanism classes and exemplars, and Figure 1B provides a simplified visual. In brief: AMR persists through interacting genetic, molecular, ecological, and cross-sector forces. Consolidating terminology around Figure 1B and Table 1 reduces redundancy while preserving the link from foundations to diagnostics, stewardship, and One Health surveillance. Figure 1A maps the One Health context with the clinical microbiologist at the center, and Figure 1B summarizes the core microbial mechanisms referenced throughout Section 2.

## 3 Antimicrobial classes, AWaRe, and stewardship guidance

This section orients the reader to major antimicrobial classes across human, veterinary, and agricultural use and links them to Access–Watch–Reserve (AWaRe). Core classes: *β*-lactams (penicillins, cephalosporins, carbapenems, and β-lactam/β-lactamase inhibitor combinations), aminoglycosides, tetracyclines, macrolides/ketolides, fluoroquinolones, polymyxins, glycopeptides (e.g., vancomycin), oxazolidinones (e.g., linezolid), and lipo−/lipopeptides (e.g., daptomycin). AWaRe groups—Access, Watch, Reserve—prioritize first-line options and stewardship oversight, with a WHO target that ≥60% of national consumption be Access (tracked through AWaRe and Essential Medicines Lists; WHO, 2022b; WHO, 2023a; WHO, 2023d). Use keeps climbing: from 2016 to 2023, estimated human consumption rose 16.3% [29.5 to 34.3 billion defined daily doses (DDDs)], reinforcing the need to shift toward Access and curb unnecessary Watch/Reserve exposure; large shares in food-animal production also persist (Van Boeckel et al., 2015; Klein et al., 2024).

For difficult-to-treat Gram-negatives, current guidance (practice-ready) covers ESBL- and AmpC β-lactamase (AmpC)-producing *Enterobacterales*, carbapenem-resistant *Enterobacterales* (CRE), DTR *P. aeruginosa*, carbapenem-resistant *A. baumannii* (CRAB), and *Stenotrophomonas maltophilia*. Preferred and alternative regimens are anchored in 2024 IDSA recommendations and complemented by European Society of Clinical Microbiology and Infectious Diseases (ESCMID) guidance standardizing empiric-to-definitive choices, enabling timely de-escalation, and protecting Reserve-class agents (Paul et al., 2022; IDSA, 2024; Tamma et al., 2024).

## 4 Diagnostic microbiology: detection and surveillance

Rapid, accurate diagnosis is central to AMR control. Culture-based methods remain the gold standard for isolation and susceptibility testing but are slow (often 48–72 h), pushing clinicians toward empiric therapy that may be off-target and resistance-promoting. Newer tools deliver faster, more precise answers, yet uptake is uneven across settings. The practical challenge is to translate speed into safer prescribing and stronger surveillance everywhere.

### 4.1 Role of modern diagnostics

Modern platforms have reshaped practice. Culture stays essential but can miss fastidious organisms and subtle mechanisms, especially in polymicrobial or biofilm-linked infections (Oliva et al., 2021). MALDI-TOF MS compresses time-to-identification to minutes after colony growth and improves outcomes in bloodstream infection and sepsis when embedded in workflows (Singhal et al., 2015; Chen et al., 2021; Torres-Sangiao et al., 2021; Elbehiry et al., 2022; Li et al., 2022). This section focuses on performance, workflow integration, and stewardship impact (mechanism definitions: Figure 1B; Table 1).

Molecular methods add both speed and depth to resistance detection. PCR identifies key marker genes such as *mecA*, *blaKPC*, *blaNDM*, and *vanA* directly from clinical specimens; multiplex panels and microarrays perform reliably for Gram-negative pathogens, and WGS often serves as the reference standard (Brazelton de Cardenas et al., 2021). WGS clarifies resistance mechanisms, tracks clonal spread, and resolves outbreaks, while tools such as ResFinder and the Comprehensive Antibiotic Resistance Database (CARD) enable high-throughput prediction of resistance determinants (Zankari et al., 2012; Alcock et al., 2020). Persistent challenges include cost, turnaround time, and limited bioinformatics capacity (Tagini and Greub, 2017).

Emerging approaches are closing key gaps. Phage-amplification assays detect viable bacteria with limits of detection as low as 20 to 40 colony-forming units per milliliter in food matrices (Phothaworn et al., 2024). Digital PCR provides absolute quantification with greater sensitivity than quantitative PCR, which is valuable for wastewater AMR surveillance and low-abundance targets (Trouchet et al., 2025). Single-cell genomics resolves the resistome and mobilome at the level of individual cells, revealing mobile elements that bulk metagenomics can miss (Kawano-Sugaya et al., 2024; Li et al., 2025). Adoption, however, is inconsistent.

Clinical impact depends on pairing rapid tests with antimicrobial stewardship programs (ASPs). Across bloodstream infection cohorts, combining rapid identification/panels with real-time stewardship shortens time-to-effective therapy, reduces unnecessary broad-spectrum use, and improves outcomes (Timbrook et al., 2016; Beganovic et al., 2019; Puckett et al., 2021). Economic analyses show these bundles are cost-effective; for bloodstream infections, MALDI-TOF integrated with an antimicrobial stewardship program (ASP) outperforms conventional workflows on costs and outcomes (Pliakos et al., 2018). Biomarker algorithms (procalcitonin) safely shorten treatment and complement rapid identification and antimicrobial susceptibility testing (ID/AST; Schuetz et al., 2018; Voermans et al., 2019).

Practical steps can accelerate impact: link rapid blood-culture identification with real-time review by the ASP to shorten time to effective therapy and curb broad-spectrum exposure (Timbrook et al., 2016); use procalcitonin to guide antibiotic discontinuation in acute respiratory infections (Schuetz et al., 2018); and build cumulative antibiograms using tools such as WHONET from routine bacteriology early in the data cycle to set locally appropriate empiric policies.

### 4.2 Surveillance systems

Laboratory data are the backbone of AMR surveillance. Over the past decade, international networks have harmonized collection across human, animal, and environmental domains. GLASS standardizes reporting and enables cross-country comparison; in Europe, EARS-Net provides extensive datasets for priority pathogens (e.g., *E. coli*, *K. pneumoniae*, *Staphylococcus aureus*), while US NARMS tracks resistance in foodborne bacteria across humans, animals, and retail meats—an explicit One Health model (Organization, W.H, 2022; EFSA/ECDC, 2024; Robillard et al., 2024).

Surveillance must feed action. Integration with ASPs turns signals into empiric-therapy guidance, cumulative antibiograms, and infection-prevention steps—now boosted by digital dashboards and real-time analytics (Crabtrey, 2021). The growing adoption of digital dashboards and real-time analytics has further empowered stewardship teams to detect resistance trends promptly and intervene at both hospital and national levels. As of 2024, GLASS reported participation by 130 countries (104 submitting national AMR data) with expanded bloodstream-infection coverage; EARS-Net and NARMS maintain public dashboards translating lab trends into policy-relevant insights across human, animal, and food chains (ECDC, 2024; WHO, 2025b). Closing the loop with hospital antibiograms ensures national signals inform bedside decisions and local infection prevention and control (IPC).

Implementation snapshots show how data can drive action. Stepwise bacteriology with WHONET in a Gambian hospital produced actionable antibiograms and guided empiric policies within the first cycle (Darboe et al., 2023). Across seven countries, an automated AMR reporting proof of concept converted routine laboratory data into standardized outputs at low marginal cost, speeding feedback for stewardship (Lim et al., 2020). In practice, epidemiologists set early-warning thresholds, veterinary services translate signals into farm-to-fork controls, and policymakers embed indicators into reporting systems, formularies, procurement, and environmental permits.

### 4.3 Gaps in laboratory capacity

Large capacity gaps persist, especially in LMICs. Fewer than 30% of facilities in parts of sub-Saharan Africa and Asia have routine access to culture and susceptibility testing (Okeke et al., 2005). Reagent shortages, staffing limitations, infrastructure constraints, and weak quality systems compound the problem (Okomo et al., 2019). Decentralization and point-of-care testing offer potential solutions. Isothermal amplification, CRISPR-based detection, and microfluidic platforms show promise for rapid, low-infrastructure identification of resistant organisms, though validation, scale-up, and regulation remain significant hurdles (Chakraborty, 2024; Hassan et al., 2025). Progress depends on sustained investment in laboratories, workforce development, quality assurance, and supply chains, alongside digital reporting to extend reach in resource-constrained settings. Strengthening diagnostics is essential for improving patient care, building robust surveillance, and advancing effective stewardship.

Practical priorities in capacity-limited settings include building stepwise ASPs aligned with the CDC Core Elements with clear diagnostic stewardship; implementing external quality assessment and standardized annual cumulative antibiograms to guide empiric therapy; deploying low-infrastructure rapid tests such as isothermal amplification or CRISPR within predefined clinical pathways; and using lightweight dashboards to deliver near real-time results to clinicians and public health teams. Even minimal infrastructure, when designed effectively, can support timely treatment, generate reliable data, and drive actionable policy (CDC, 2018; CDC, 2022).

**Figure 2:**
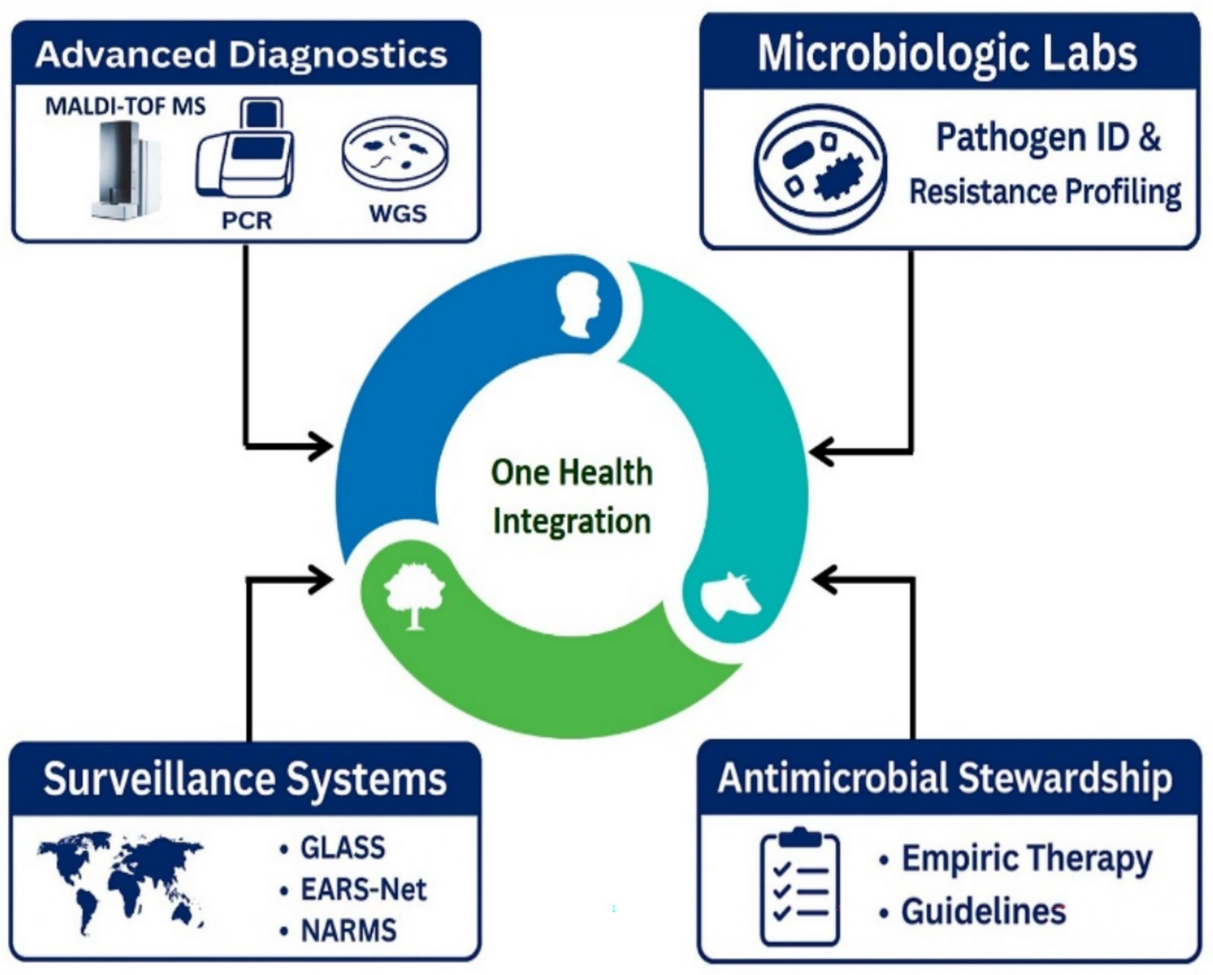
Diagnostic pipeline integrating AMR control within a One Health framework. Rapid platforms such as MALDI-TOF MS, PCR, and WGS feed pathogen identification and resistance profiling into the microbiology laboratory, where results aggregate into cumulative antibiograms and guide antimicrobial stewardship programs for empiric selection and timely de-escalation. Aggregated laboratory signals flow to GLASS, EARS-Net, and NARMS to track regional and global resistance trends, closing the loop between diagnostics, stewardship, surveillance, and policy. This is a schematic overview, and icons are not to scale. Device-specific parameters are intentionally omitted to emphasize workflow integration and clinical impact. Cross-sector transmission pathways are shown in Figure 3.

Figure 2 provides an overview of the diagnostic pipeline, showing how MALDI-TOF MS, PCR, and WGS connect to laboratory-generated antibiograms, empiric therapy guidance, and ASPs. These data then feed into global and regional surveillance systems such as GLASS, EARS-Net, and NARMS, creating alignment between diagnostics, stewardship, and surveillance within a One Health framework and positioning microbiologists at the critical interface of laboratory science, clinical decision-making, and policy.

## 5 Antimicrobial stewardship: strategies and innovations—bridging diagnostics to action

Rapid diagnostics have the greatest impact when tied to active stewardship. Meta-analyses and multicenter studies show that pairing molecular or MALDI-TOF–based ID/AST with real-time pharmacist or infectious-diseases input shortens time-to-effective therapy, reduces broad-spectrum exposure, and can lower mortality in bloodstream infection. Practical levers include targeted/cascade reporting, diagnostic stewardship (clear “when to test/when not to test”), and routine prescriber feedback anchored in local antibiograms and national guidance (CDC Core Elements; IDSA/SHEA). Embedding these elements converts lab speed into timely prescribing changes and measurable outcomes (Timbrook et al., 2016; Beganovic et al., 2017; Wolk et al., 2025).

### 5.1 Global disparities

Worldwide, inpatient antibiotic use remains high and skewed toward broader-spectrum Watch agents: in pooled point-prevalence surveys, prescribing is often >50%, with Watch antibiotics meeting or exceeding Access in many LMICs (Pauwels et al., 2021; Boltena et al., 2024). Against stewardship targets, AWaRe calls for ≥60% Access consumption, and the 2024 UN High-Level Meeting urged progress toward 70% by 2030; recent compilations show many countries remain below these thresholds (WHO, 2023a; CIDRAP, 2025). A 2025 review of 85 studies found only ~14% met the 70% Access benchmark, with overuse of Watch antibiotics reported in ~68%—underscoring persistent imbalance (Saleem et al., 2025). While programs reduce total use, robust impact evaluations and implementation resources remain scarce in resource-constrained settings—arguing for standardized indicators, routine antibiograms, and practical audit/feedback models tailored to local capacity.

### 5.2 Clinical stewardship programs

The most effective antimicrobial stewardship programs are multidisciplinary, involving infectious diseases clinicians, clinical microbiologists, pharmacists, infection preventionists, and data analysts. Core strategies include formulary restriction, prospective audit with feedback, antibiotic “time-outs,” and use of cumulative antibiograms to refine empiric therapy (Barlam et al., 2016). Microbiologists play a central role by providing rapid organism identification and resistance profiles that enable earlier treatment adjustments. Evidence shows that well-implemented programs reduce inappropriate antibiotic use, improve patient outcomes, and lower rates of *Clostridioides difficile* (*C. difficile*) infection (MacDougall and Polk, 2005; Dellit et al., 2007). For example, integrating microbiology data with real-time stewardship feedback reduced broad-spectrum antibiotic use by 25% in a multicenter US study (Pollack et al., 2016).

Operational snapshots show how coordinated workflows change practice. In one consortium, linking rapid identification and susceptibility testing to pharmacist and microbiologist callbacks reduced broad-spectrum days of therapy, shortened time to effective treatment, and increased de-escalation within 48 to 72 h, driven by targeted and cascade reporting aligned with local antibiograms (Banerjee et al., 2015; MacVane and Nolte, 2016; Peri et al., 2024). In LMICs, implementation is constrained by limited microbiology infrastructure, weak policy enforcement, and high prescriber autonomy (Laxminarayan et al., 2013; Charani et al., 2024). WHO toolkits encourage context-sensitive approaches tailored to local epidemiology and resources, emphasizing diagnostic stewardship, standardized reporting, and embedding indicators into accreditation processes (Organization, W.H, 2019).

### 5.3 Decision support tools and artificial intelligence

Clinical decision support systems (CDSSs) integrate patient data (comorbidities, allergies, renal function, and prior cultures) with local antibiograms and guidelines to generate recommendations at the point of care (Livorsi et al., 2015). AI models extend this by predicting resistance phenotypes from genomic/phenotypic inputs; machine-learning approaches can infer susceptibility from WGS (Nguyen et al., 2018; Hicks et al., 2019) and forecast AMR outbreaks across hospital networks (Rawson et al., 2017; Straw, 2020). Electronic prescribing with stewardship modules improves accuracy and reduces errors, and linkage to laboratory information systems creates real time feedback loops (Hamdan et al., 2024). The strength of AI is its ability to operate at scale by fusing clinical, laboratory, epidemiologic, and genomic data (Abdelwanis et al., 2024; Howard et al., 2024). Guardrails such as human oversight, interpretable models, and ongoing training are essential. Technology amplifies good workflow; it does not replace it.

### 5.4 Education and behavior change

Behavior change is the backbone of stewardship. Knowledge gaps, fear of undertreatment, and patient expectations drive misuse (Hulscher et al., 2010; Charani et al., 2013). Effective programs use interactive case discussions, peer comparison, audit-and-feedback, and behavioral nudges. Public commitment posters reduce unnecessary prescribing in outpatient care (Meeker et al., 2014). In hospitals, 48–72 h antibiotic time-outs improve de-escalation and discontinuation (Lynch et al., 2025). Community education matters: misconceptions about antibiotics for viral illness, self-medication, and OTC access remain pervasive; national campaigns (e.g., “Get Smart,” “Antibiotic Guardian”) help shift norms (Huttner et al., 2010; McNulty et al., 2013).

Practical strategies in outpatient and primary care are straightforward and reproducible. Public commitment posters and delayed prescriptions help manage self-limiting respiratory tract infections, while monthly peer-comparison reports combined with brief audit and feedback targeting respiratory and urinary syndromes encourage more appropriate prescribing. Pharmacy-based counseling with clear scripts such as “no antibiotics needed” or “expected course” supports patient understanding of viral illnesses, and partnerships with national campaigns help reinforce community norms (McNulty et al., 2013; Meeker et al., 2014; Bhattacharya et al., 2017). A proven model is Thailand’s Antibiotics Smart Use program, which reduced unnecessary prescribing by combining clinician and patient commitment devices with condition-specific interventions—an adaptable template for primary care in LMICs (Sumpradit et al., 2012; Boonyasiri and Thamlikitkul, 2014; Waleekhachonloet et al., 2021).

Stewardship achieves its greatest impact when education is paired with system levers default prescription durations, restriction policies, and stewardship-linked electronic order sets. Ultimately, success depends on adaptability to local epidemiology and integration across human, veterinary, and environmental sectors. Stewardship is a dynamic strategy that blends coordinated clinical programs, innovative decision-support, and robust behavior change. In high-income settings, rapid diagnostics and digital platforms amplify these gains; in LMICs, progress hinges on investment in infrastructure, regulation, and workforce. Across all contexts, microbiologists remain pivotal in translating laboratory evidence into actionable guidance that connects diagnostics, stewardship, and policy.

Community and culture shape prescribing through clinician risk aversion and hierarchy, patient expectations, and, especially in many LMICs, ready over the counter access and informal markets that normalize self-medication. Effective programs pair interactive teaching and audit and feedback with brief “no antibiotics needed” scripts, public commitment displays, and peer comparison dashboards, while pharmacy-based counseling reduces inappropriate demand and supply (Hulscher et al., 2010; Charani et al., 2013; Meeker et al., 2014; Bhattacharya et al., 2017). A practical LMIC blueprint focuses on five community syndromes (acute respiratory infection, otitis media, pharyngitis, diarrheal disease, cystitis) using one page decision trees; issues simple cumulative antibiograms with quarterly audit and feedback to clinics and pharmacies; deploys commitment posters and brief local language counseling scripts; engages community health workers for education and adherence follow up; and, where feasible, curbs over the counter sales through professional and pharmacy associations and regulators (Hulscher et al., 2010; Charani et al., 2013; Adhikari et al., 2021).

**Figure 3:**
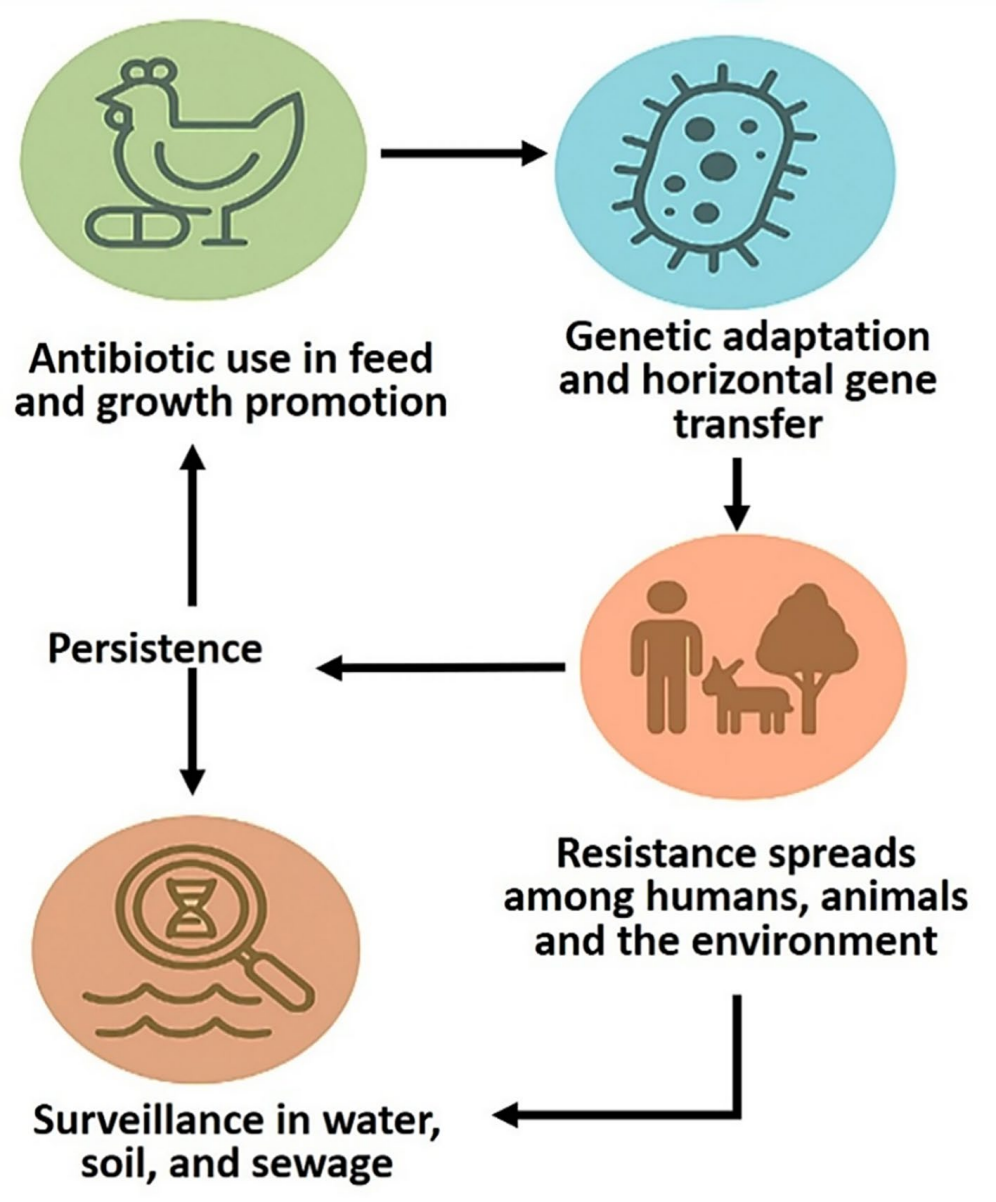
One Health model of AMR emergence and spread. Antibiotic use in livestock and agriculture selects resistant bacteria that evolve through mutation and horizontal gene transfer, then move between humans, animals, and the environment via food, direct contact, and ecological pathways. Persistence in soil, water, and sewage amplifies the cycle, while surveillance in these reservoirs provides early-warning signals that can be routed back to bedside and policy. Single reference figure for One Health pathways; mechanism terms align with Figure 1B and Table 1. Climate modifiers—temperature, extreme rainfall, hydrologic change can accelerate these routes; event-based sampling and catchment triggers turn signals into timely clinical and public-health responses.

Socioeconomic drivers remain central, and pairing brief, behavior-informed education with national campaigns such as Antibiotic Guardian has proven effective in shifting norms and prescribing patterns (Bhattacharya et al., 2017; Adhikari et al., 2021). Program design, data and AI, and behavior change strategies work best in combination, and Figure 3 illustrates this integrated stewardship model while linking it to the broader One Health framework.

## 6 One health integration and environmental dimensions

The global response to antimicrobial resistance now recognizes that human, animal, and ecosystem health are inseparable. The One Health framework, endorsed by the WHO, the Food and Agriculture Organization (FDA), the World Organization for Animal Health (WOAH), and the United Nations Environment Program (UNEP), aligns bedside decisions with upstream drivers and downstream exposures and links surveillance signals to coordinated action across sectors (WHO, 2022a). To avoid repetition, cross-sector pathways (food chain, wastewater, aquaculture, wildlife, co-selectors) appear once in Figure 3; mechanism terms follow Figure 1B and Table 1.

### 6.1 AMR in agriculture and livestock

Antibiotics in livestock—therapeutic, prophylactic, and growth-promotion remain major engines of selection; >70% of global consumption occurs in food-producing animals (Van Boeckel et al., 2015). Heavy use of tetracyclines, sulfonamides, and macrolides drives MDR organisms, including ESBL-producing *E. coli*, *Salmonella enterica*, and *Campylobacter* spp. (Madec et al., 2017; Tang et al., 2017). Zoonotic spillover is well documented: identical genes and plasmids in *E. coli* from livestock, retail meat, and human infections (Liu et al., 2016; Nhung et al., 2016). Manure-borne residues fertilize fields, intensifying selection and HGT in soil microbiota (Zhu et al., 2013). Mitigation—EU growth-promoter bans, veterinary consumption monitoring, and non-antibiotic alternatives—shows promise, but enforcement is uneven in LMICs (More, 2020).

Crop protection (e.g., streptomycin, oxytetracycline) adds risk, prompting recent U. S. actions (EPA, 2024). Practical links to clinical care include routine comparative genomics of priority determinants/plasmid backbones across livestock–meat–patient isolates and pre-season veterinary–clinical antibiogram exchanges to anticipate food-chain risks; EU One Health surveillance tracks zoonotic AMR along the chain (Ludden et al., 2019; EFSA/ECDC, 2025). Policy success is instructive: after discovery of plasmid-mediated colistin resistance (*mcr-1*), China’s 2017 feed ban was followed by declines in *mcr-1* carriage and phenotypic resistance across animals, food, and humans (Shen et al., 2020; Wang et al., 2020; Shen et al., 2021).

### 6.2 Environmental pathways of resistance

Antibiotics, resistant bacteria, and ARGs enter ecosystems through hospital and pharmaceutical effluents, agricultural runoff, wastewater, and landfills, creating environmental reservoirs where resistance can evolve and spread (Berendonk et al., 2015; Larsson et al., 2018). Elevated drug levels and enriched resistance determinants have been detected downstream of pharmaceutical manufacturing plants (Larsson et al., 2007). Wastewater treatment plants (WWTPs) often fail to completely remove antibiotics or resistant microbes, releasing mobile genetic elements into rivers and coastal waters (Rizzo et al., 2013; Karkman et al., 2018). Aquaculture further contributes as a hotspot of direct selection within aquatic ecosystems (Cabello et al., 2013). Surveillance remains fragmented, particularly in LMICs, and integration of clinical and environmental data is still limited (Hendriksen et al., 2019).

Operational solutions include monitoring the wastewater and catchment resistome so that spikes in priority genes or mobile elements trigger hospital screening, hygiene audits, and reviews of empiric therapy; feasibility has been demonstrated by global sewage metagenomics and WHO’s Tricycle ESBL-producing *E. coli* track (Hendriksen et al., 2019; WHO, 2021). Reuse irrigation and wildlife further extend spread, as treated wastewater irrigation enriches antimicrobial resistance genes along soil, plant, and invertebrate interfaces, while gulls and other birds disseminate ESBL-producing *E. coli* over long distances (Zeballos-Gross et al., 2021; Fuentes-Castillo et al., 2023; Bhattacharjee et al., 2024). Climate pressures amplify these flows, because heat waves and storm events increase loads and transport, which supports event-based sampling and river network triggers linked to hospital and veterinary dashboards (MacFadden et al., 2018; Tipper et al., 2024).

### 6.3 Microbiologists’ role in environmental AMR mapping

Microbiologists connect environmental surveillance to clinical and veterinary action. Metagenomics and resistome profiling detect resistance determinants in complex communities and reveal novel diversity (Martínez et al., 2015; Ghaly et al., 2017). Population scale wastewater monitoring is feasible and cost effective for detecting urban trends (Hendriksen et al., 2019). This approach offers a cost effective, noninvasive strategy for tracking AMR, especially in cities and regions with limited clinical infrastructure. Effective control requires shared governance: epidemiologists co-design sampling and attribution; veterinary and environmental agencies lead farm, food chain, and catchment interventions; and policymakers translate triggers into reporting, purchasing, reimbursement, and discharge rules aligned with quadripartite One Health guidance and GLASS One Health tracks (FAO, 2022; EFSA/ECDC, 2025). A practical operating model aligns sample frames (clinical isolates, veterinary pathogens, sewage and surface waters) and shared metadata (AMU/AMC, exposure, geography) in unified dashboards with predefined triggers for joint responses such as food chain investigations, wastewater remediation, and formulary review.

Figure 3 depicts One Health emergence and dissemination: agricultural use selects resistant bacteria that reach humans via contact, food, and environmental routes; wastewater, pharmaceutical effluents, and runoff seed soil and waters; and microbiologists sit at the center, linking resistome mapping, metagenomic surveillance, and data integration to inform practice and policy. Table 1 synthesizes key drivers, representative genes, and intervention points, highlighting how integrated surveillance and stewardship can contain AMR at its ecological roots.

## 7 Translational and policy challenges

Scientific progress is not the same as policy progress. Despite advances in detection, surveillance, and stewardship, action often stalls because of fractured regulations, weak cross sector coordination, and poor incentives for developing new antibiotics. Closing this gap requires wiring laboratory signals to decisions through explicit triggers, such as spikes in bloodstream infection resistance or outlier Standardized Antimicrobial Administration Ratio (SAAR) values, and through routine dashboards that convert data into regulatory actions, formulary updates, and purchasing decisions. WHO’s, 2023 monitoring and evaluation guidance for AMR National Action Plans (NAPs) provides indicators and reporting cycles that ministries can embed in law and budgets, and the National Healthcare Safety Network Antimicrobial Use and Resistance module shows how standardized antimicrobial use and resistance reporting can support stewardship oversight (WHO, 2023c; NHSN, 2025). The next subsections outline where policy fails, who needs to be at the table, why stewardship uptake diverges across settings, and how incentives can reward genuine innovation without encouraging overuse.

Measure success with practical indicators that tie directly to action: AWaRe targets for Access antibiotics at 60% now and moving toward 70%, days of therapy per 1,000 patient days, time to effective therapy, GLASS completeness, stockout and continuity days, and compliance with antibiotic manufacturing effluent standards (WHO, 2023c, 2025a; WHO, 2022b; WHO, 2023c; NHSN, 2025; CLSI, 2022). These metrics should be reported routinely, displayed on simple dashboards, and reviewed in standing stewardship and quality meetings.

AMR is ultimately systemic: discovery, pricing, and use are shaped by markets and rules. Because short treatment courses and stewardship limit revenue, delinked incentives and subscription models are needed to reward access and clinical impact rather than sales volume (Outterson and Rex, 2016). Procurement can also steer behavior by aligning national formularies with AWaRe, promoting appropriate first line choices (WHO, 2022b). In many LMICs, weak regulation and informal markets sustain over-the-counter sales (Sakeena et al., 2018), while unmanaged effluents seed ecosystems with active compounds (Larsson et al., 2007). Decades of non-therapeutic use in livestock/aquaculture spread resistance across borders though EU growth-promoter bans and China’s colistin restrictions show policy can bend the curve (Van Boeckel et al., 2015; Shen et al., 2020). In brief: market reform, smarter procurement, enforceable regulation, and environmental safeguards must travel with biomedical innovation.

### 7.1 Regulatory inconsistencies and enforcement gaps

Rules vary widely across countries. High income settings often mandate prescribing controls and veterinary regulation with strong surveillance, while many low and middle income countries lack enforceable policies, funded National Action Plans, or restrictions on over the counter sales (Rochford et al., 2018; Sakeena et al., 2018; Sulis et al., 2022). Resistance data are frequently siloed, limiting real time response (Ruckert et al., 2024). Aligning legal instruments with microbial surveillance data remains a critical unmet need in AMR governance. Practical fixes include requiring antimicrobial use and resistance reporting through GLASS and the NHSN AUR for licensure or reimbursement, enforcing bans on over the counter sales with inspections, publishing facility level dashboards, and appointing a national AMR lead with cross sector authority (WHO, 2023c; NHSN, 2025).

Only about 11% of countries report dedicated budgets for NAPs, and although the Quadripartite platform launched in November 2022 aims to improve coordination, alignment of legal instruments with GLASS and national monitoring and evaluation remains uneven (WHO, 2022c, 2023c). A system lesson from England shows how surveillance plus targeted interventions can shift practice: mandatory reporting of methicillin resistant *Staphylococcus aureus* bacteraemia, combined with infection prevention and stewardship initiatives under the Health Act 2006 and the cleanyourhands campaign, coincided with sustained reductions (Johnson et al., 2012; Stone et al., 2012).

### 7.2 Engaging microbiologists in policy and advocacy

Microbiologists hold critical signals yet are often absent from policy discussions, leading to missed trends and underused laboratory capacity (Gandra et al., 2020). Embedding them in governance as advisors on national task forces and within the Global Leaders Group ensures that microbial data inform risk assessment, priority setting, and use restrictions (Mattar et al., 2020). Beyond surveillance, they can contribute to health technology assessments and cost–benefit analyses and add credibility to public messaging, as seen with India’s Red Line and the UK TARGET Toolkit (Chokshi et al., 2019). Two practical enablers are protected time and funding for roles embedded in ministries and National Action Plan secretariats, and data sharing memoranda of understanding that move de-identified laboratory data into public health dashboards (WHO, 2023c). Structured avenues already exist, including the Global Leaders Group, India’s Red Line, and the UK TARGET Toolkit, which link laboratory signals to tools that shift prescribing (Travasso, 2016; Mathew et al., 2022).

### 7.3 Disparities in stewardship uptake

Stewardship remains uneven. High income systems benefit from mandates, electronic prescribing, and dedicated teams, whereas LMICs face limited diagnostics, unregulated distribution, and workforce gaps (Tattevin et al., 2020). In 10 sub-Saharan hospitals, only 20% had updated antibiograms and less than 30% conducted regular audits (Cox et al., 2017). WHO’s LMIC toolkit promotes modular steps such as leadership, simple indicators, and point of care education (Maki et al., 2020). A minimum starter package includes one CLSI M39 compliant cumulative antibiogram per year, a 48 to 72 h antibiotic time out, and a monthly audit and feedback cycle for pneumonia, urinary tract infection, and sepsis supported by a one page dashboard (WHO, 2019; CLSI, 2022; CDC, 2025). Meta analyses and surveys show that inpatient antibiotic use remains high at about 60% and that formal hospital stewardship programs are sparse; where implemented, these programs improve appropriateness and reduce unnecessary use (WHO, 2019; Simner et al., 2022; Gobezie et al., 2024). Finally, stewardship must align with incentives, and the next subsection outlines delinked payment models that protect access while preserving efficacy.

### 7.4 Incentivizing antibiotic innovation without overuse

Traditional markets under-reward antibiotic R&D because short treatment courses and restricted use translate into limited revenues (Renwick et al., 2016). Solutions include market entry rewards, delinked payments, and subscription contracts (Outterson et al., 2016). Implementation is advancing: England launched subscription contracts in 2022 for cefiderocol and ceftazidime-avibactam after a bespoke National Institute for Health and Care Excellence evaluation (NICE) that valued access rather than sales (NICE, 2022; Woods et al., 2025). In May 2024, NHS England issued national guidance enabling UK-wide participation, backed by about £100 million in 2024/25 funding (NHS, 2024; Parmaceutical-Journal, 2024). In the United States, the bipartisan PASTEUR Act would authorize federal subscriptions for “critical need” agents (Congress.GOV, 2023). Pull incentives must also carry access and stewardship conditions: in 2019, only about 6.9% of patients with carbapenem-resistant Gram-negative infections across eight large LMICs received an appropriate agent (GARDP, 2025; Mishra et al., 2025). Contracts should require supply security, global access clauses, and enforceable effluent standards, and they should be evaluated with transparent metrics such as access days saved, mortality averted, and preserved susceptibility, with periodic recalibration (NICE, 2022; NHS, 2024; WHO, 2024a). Table 2 summarizes these barriers and highlights how microbiologists can help move from data to decisions by aligning surveillance, stewardship, and policy design so that innovation is rewarded without driving overuse.

**Table 2:** Translational and policy challenges in AMR control and the role of microbiologists.

Challenge area	Description of the barrier	Implications for AMR control	Role of microbiologists	Key references
Regulatory inconsistencies & enforcement gaps	Wide variation in AMR regulation and enforcement; many national action plans incompletely funded/implemented; siloed data systems.	Fragmented governance and slow/uneven policy response; weak linkage of surveillance data to decisions.	Provide standardized lab data and analytics into national AMR plans; contribute to harmonized indicators and GLASS reporting.	WHO (2015, 2024b)
Limited engagement of microbiologists in policy & advocacy	Lab evidence often remains within clinical/academic circles; advisory fora underused at country level.	Policies may miss resistance trends or underuse lab capacity; weaker public communication.	Serve on national AMR task forces/GLG-linked fora; translate lab data into risk assessments/guidelines; contribute to public campaigns.	Nair et al. (2021); Sparrow et al. (2024)
Disparities in stewardship uptake	ASPs robust in many HICs but variable in LMICs; gaps in diagnostics, audits, and workforce.	Overuse/misuse persists; delayed containment of resistant strains.	Generate antibiograms; lead audit/feedback; tailor guidelines to local epidemiology and resources.	WHO (2019); Siachalinga et al. (2023)
Innovation paradox & weak R&D incentives	Traditional market models discourage antibiotic R&D; pipeline remains limited for critical MDR threats.	Thin pipeline; risk of over- or under-use without access/stewardship safeguards.	Prioritize targets from surveillance; support HTA inputs; co-design deployment with stewardship.	WHO (2024a)

### 7.5 LMIC-ready operations toolkit: affordable, scalable steps from surveillance to action

Equity is achievable with a lean package. Start with a district level diagnostic core: stepwise bacteriology (blood, urine, and stool culture; Kirby Bauer disk diffusion) to EUCAST and CLSI standards and a test menu aligned with the WHO Essential Diagnostics List, which keeps capital needs low and interoperability with national and global surveillance high (WHO, 2023b; EUCAST, 2025). Make cultures count with WHONET, license free software that cleans AST data, generates CLSI M39 compliant antibiograms, and exports to GLASS on a standard laptop (WHO, 2021; WHONET, 2025). Stewardship can scale without hardware: a small multidisciplinary team implements 48 to 72 h time outs, simple audit and feedback, and antibiogram guided empiric pathways using the CDC Core Elements and WHO’s LMIC toolkit, with OpenWHO courses accelerating training (WHO, 2021; WHONET, 2025).

Community levers amplify impact. Thailand’s Antibiotics Smart Use shows that commitment devices plus condition specific guidance reduce unnecessary antibiotics, and low cost point of care C reactive protein testing reduced antibiotics for non-severe respiratory infections in Vietnam (Sumpradit et al., 2012; Do et al., 2016). A simple One Health stream ties signals to action: a “Tricycle lite” model samples quarterly across humans, poultry, and sewage; tracks an ESBL *E. coli* indicator with basic culture plus targeted qPCR (e.g., *blaCTX-M*, *mcr*, *intI1*); and uses predefined triggers (a twofold rise in ESBL *E. coli* or first detection of *mcr*) to launch paired responses—hospital screening and hygiene audits upstream, environmental controls downstream (Do et al., 2016; WHO, 2019; CLSI, 2022; Do et al., 2023). Track a minimal indicator set—percent Access (AWaRe), DOT per 1,000 patient days, time to effective therapy, antibiogram coverage, and laboratory turnaround—to demonstrate value and guide scale up(CLSI, 2022; WHO, 2023a; WHO, 2023c; NHSN, 2025).

## 8 Future directions and research gaps

Despite growing awareness and substantial progress in AMR research, numerous scientific, technical, and policy challenges remain unresolved. Addressing these gaps requires bold, interdisciplinary strategies that span microbial evolution, diagnostic innovation, translational barriers, and ecological persistence. This section highlights priority research directions and emerging approaches that could transform the AMR landscape over the next decade.

### 8.1 Biomarker-guided antibiotic therapy

Host response markers such as procalcitonin, C-reactive protein, and gene expression signatures can help distinguish bacterial from viral illness and reduce unnecessary antibiotic exposure (van Houten et al., 2019). In a multicenter trial, procalcitonin-guided discontinuation safely reduced antibiotic use for respiratory infections (Schuetz et al., 2013). The challenge is validation, because tools that perform well in development often falter on external testing, including sepsis models with declining AUROC on out-of-sample data (Yusuf et al., 2024; Wang et al., 2025). Validation should be treated as a prerequisite rather than a formality. Hybrid platforms that pair pathogen detection with host profiling, such as the 29-mRNA InSep test, show promise in emergency care for targeting therapy (Safarika et al., 2021).

### 8.2 CRISPR-based antimicrobial strategies

Programmable CRISPR payloads can knock out resistance genes and resensitize bacteria, as shown in early studies against multidrug-resistant *E. coli* and *β*-lactamase and carbapenemase targets in Enterobacterales (Citorik et al., 2014; Yosef et al., 2015; Ahmed et al., 2024; Rafiq et al., 2024). Clinical signals are emerging but still preliminary: a CRISPR-Cas3–enhanced phage cocktail (LBP-EC01) reported Phase 2 Part 1 safety and target engagement in uncomplicated *E. coli* urinary tract infection, and SNIPR001 advanced with supportive animal data (Gencay et al., 2024; Kim et al., 2024). Key bottlenecks include reliable delivery, bacterial escape, host responses, off-target effects, and GMP scalability, all within regulatory pathways that remain nontrivial (Fage et al., 2021; Sioson et al., 2021; Mayorga-Ramos et al., 2023; FDA, 2025a, 2025b). A practical stance is to frame CRISPR antimicrobials as promising complements awaiting randomized trials, standardized manufacturing, and harmonized guidance, with environmental safeguards such as kill switches and containment and with equity built in (de la Fuente Tagarro et al., 2024). Ethical, ecological, and sustainability guardrails are essential: clinical application should proceed only after environmental and biocontainment risk assessments are completed in advance, using nonreplicating or self-limiting vectors where appropriate, followed by post-deployment surveillance, and should align with antimicrobial stewardship and long-term monitoring for resistance development.

### 8.3 Global surveillance linking clinical and environmental isolates

Clinical systems capture only part of the picture. Wastewater metagenomics shows clear overlap between hospital effluents and downstream waters, confirming incomplete removal and persistent environmental reservoirs (Su et al., 2024) Population scale sewage surveillance is feasible and cost effective (Hendriksen et al., 2019). The next step is an interoperable One Health database that fuses environmental resistomes with clinical outcomes to provide early warning, support risk attribution, and guide targeted interventions.

### 8.4 Microbiome-aware antibiotic development

Microbiome sparing therapy can reduce recurrence and collateral damage. Ibezapolstat, a DNA polymerase IIIC inhibitor for *C. difficile* infection, has shown activity against resistant strains while preserving key taxa (Bassères et al., 2024) Development pipelines should screen for microbiome impact early, and trials should track recovery and resistance gene carriage after treatment. Multiomics continues to reveal how antibiotics perturb networks and mobilize genes, guiding precision agents and stewardship to preserve ecological balance (Guitart-Matas et al., 2025; Lu et al., 2025). At the bedside, a staged approach prioritizes agents with lower collateral damage where guidelines support their use—for example, the IDSA focused update favoring fidaxomicin for *C. difficile* infection when resources permit—tracks simple “microbiome proxies” such as *C. difficile* recurrence and colonization with multidrug resistant organisms, and embeds formulary nudges including default durations and restrictions on highly disruptive classes (IDSA, 2021).

### 8.5 Antimicrobial peptides: promise and pitfalls

AMPs can rapidly disrupt bacterial membranes and, in some scaffolds, act on intracellular targets, producing swift killing with a relatively high barrier to single step resistance. Translation to systemic therapy, however, remains constrained by dose limiting host cell toxicity, rapid proteolysis in serum, complex PK/PD relationships, and cost intensive manufacturing (Bechinger and Gorr, 2017; Min et al., 2024)(Cresti et al., 2024; Zhang, 2025). Clinical findings are mixed: topical pexiganan has shown signals of benefit in randomized multicenter trials for diabetic foot infection, and omiganan has demonstrated pharmacodynamic activity and microbiome effects in randomized dermatologic and catheter site studies (Lipsky et al., 2008; Niemeyer-van der Kolk et al., 2020; Niemeyer-van der Kolk et al., 2022). Among host-defense–mimetic compounds, brilacidin achieved phase-2 noninferiority versus daptomycin for ABSSSI (Giacobbe et al., 2022), whereas the *Pseudomonas*-targeted peptidomimetic murepavadin (LptD inhibitor) had phase-3 programs halted due to higher-than-expected acute kidney injury (Prasad et al., 2022; Elmassry et al., 2023).

Key barriers—host cell toxicity, short half-life from proteolysis, challenging PK/PD, and manufacturing cost—are being addressed through structure and delivery engineering. D amino acid substitutions, sequence cyclization, PEGylation, and nanoformulations such as lipid or polymer nanoparticles and hydrogels aim to enhance stability, bioavailability, and tissue localization while preserving antibacterial potency (Ma et al., 2024; Min et al., 2024; Zheng et al., 2025). Resistance can still emerge through cell envelope remodeling, surface charge modification, and protease upregulation, underscoring the need for careful stewardship (Nawrocki et al., 2014; Bechinger and Gorr, 2017). In practice, stewardship for AMPs should emphasize narrowly defined indications, topical or localized delivery when feasible, combination therapy to reduce selective pressure, and rigorous PK/PD and safety evaluation before broader deployment.

Sustained progress with AMPs depends on integration rather than isolated advances. Priorities include precision diagnostics to match patients and dosing windows, programmable antimicrobials that complement peptide mechanisms, and surveillance systems capable of resolving peptide specific resistance pathways. As multiomics and microbiome sparing strategies mature, microbiologists remain central to linking bench discoveries with ecological monitoring and policy translation. The coming decade will be shaped not only by better peptide design and delivery but also by the validation, integration, and responsible application of these tools across human, animal, and environmental health.

## 9 Conclusion

AMR is a defining health threat rooted in microbial genetics, accelerated by misuse, and sustained across clinical, agricultural, and environmental systems. This review traced foundational biology, the centrality of diagnostic microbiology, the urgency of stewardship, and the necessity of a One Health lens. At the center stands the clinical microbiology laboratory: it generates the signals that power detection, surveillance, and stewardship. Those signals matter most when they trigger policy, procurement, and practice across settings, from rural clinics to wastewater networks. Near term practical path: make mechanism–organism matching routine by pairing phenotypes with targeted assays and clear reporting rules; couple rapid ID/AST to real time stewardship and cumulative antibiograms so speed becomes safer prescribing; wire laboratory feeds to early warning dashboards that trigger hospital IPC and national actions; extend monitoring across the One Health continuum, including storm responsive, catchment scale sampling, so environmental signals prompt clinical and veterinary responses; and align incentives and procurement with stewardship, using AWaRe consistent formularies, delinked or subscription payments with guardrails, and enforceable manufacturing effluent standards. Progress depends on validated, scalable tools: biomarker guided therapy, microbiome sparing drugs, CRISPR based resistance disruption, and global genomic and metagenomic surveillance, paired with sustained investment and equity, especially in LMICs. Track improvement with a compact scorecard: % Access (AWaRe), days of therapy per 1,000 patient days, time to effective therapy, antibiogram and GLASS completeness, stockouts, and compliance with effluent limits. The future of antimicrobial therapy will be secured not by discovery alone, but by how wisely, fairly, and collaboratively existing and emerging tools are deployed. The frameworks, tables, and case snapshots in this review provide step by step triggers to translate diagnostic signals into prescribing change, surveillance reporting, and regulatory action across diverse resource settings. The future of antimicrobial therapy will not be secured by discovery alone, but by our collective ability to deploy existing and emerging tools wisely, equitably, and collaboratively.
